# Comparison of Renal Blood Flow Using Maximum Slope-Based Computed Tomography Perfusion and Ultrasound Flow Probe in Healthy Dogs

**DOI:** 10.3389/fvets.2020.541747

**Published:** 2020-10-09

**Authors:** Sang-Kwon Lee, Youjung Jang, Jin-Woo Jung, Hyejin Je, Jihye Choi

**Affiliations:** Veterinary Medical Imaging, College of Veterinary Medicine and BK21 Plus Project Team, Chonnam National University, Gwangju, South Korea

**Keywords:** computed tomography perfusion, dog, maximum slope, renal perfusion, renal function, ultrasonic flow probe

## Abstract

Computed tomography (CT) perfusion can analyze tissue perfusion and quantitative parameters, including blood flow, blood volume, and transit time. CT perfusion has been used for evaluating split renal function. However, its applicability in veterinary medicine was not validated. This study aimed to evaluate the correlation of renal blood flow (RBF) derived by maximum slope-based CT perfusion and an ultrasonic flow probe and assess the effect of the presence of a pre-existing contrast medium on CT perfusion in the kidneys. In five healthy purpose-bred beagles, CT perfusion was performed at the level of the left renal hila after injection of 1 mg/kg iohexol, during measuring RBF with an ultrasonic flow probe placed on the left renal artery. After post-contrast CT scan with injection of 2 mg/kg iohexol, CT perfusion scan was repeated with the same protocol used in the first perfusion study. The CT perfusion derived RBF was analyzed based on the maximum slope and was compared with the true RBF obtained using an ultrasonic flow probe. Results indicated that CT perfusion derived RBF was significantly correlated with true RBF, although CT perfusion derived RBF did not match the absolute value of the true RBF. It was correlated with the true RBF, even in the presence of a pre-existing contrast medium in the kidney. CT perfusion can estimate the change in individual renal perfusion non-invasively, and this method can be used supplementary to the conventional CT protocol in clinic.

## Introduction

Renal blood flow (RBF) influences the regulation of changes in glomerular filtration rate and renal tissue oxygenation (1, 2). Renal hypoperfusion and associated hypoxia are key elements in the pathophysiology of acute kidney injury and progression to chronic kidney diseases (2). Renal perfusion values are measured to evaluate renal function in human patients with unilateral ureteral obstruction, split renal function in kidney transplantation donors, and the hemodynamic significance of renal artery stenosis (3–5). Renal perfusion measurements are used to detect and differentiate the different types of renal tumors and to assess the efficacy of antiangiogenic tumor treatments (5–7). In veterinary medicine, renal perfusion was evaluated in dogs and cats with acute kidney injury, chronic kidney disease, and renal ischemia-reperfusion injury and those who received kidney transplantation (8–11).

There are only few methods that can be used in the non-invasive evaluation of renal perfusion. These methods include contrast-enhanced ultrasound (CEUS), dynamic contrast-enhanced magnetic resonance imaging, and isotopic renography (4, 5, 12, 13). The absolute quantification of renal perfusion on contrast-enhanced magnetic resonance imaging is challenging to perform, because there is no linear relationship between signal intensity and the concentration of the contrast medium (12, 13). Isotopic renography can evaluate split renal function however it has limitations, such as radiation exposure and high cost (4). CEUS can evaluate renal perfusion change reproducibly and is easily used in routine clinical practice in dogs and cats (8–11). It can reflect kidney perfusion based on indirect perfusion parameters but cannot provide absolute perfusion values.

Computed tomography (CT) perfusion is based on temporal changes in tissue enhancement and analytic approaches including Patlak analysis, deconvolution, and maximum slope methods for computing perfusion values (14). CT perfusion is widely used in humans because it has better spatial resolution than magnetic resonance imaging or scintigraphy, and it facilitates the absolute quantification of renal perfusion based on the strict linear relationship between the measured renal tissue attenuation and the concentration of the contrast medium (4–6). Previous studies in pigs have demonstrated that CT perfusion values are significantly correlated with renal perfusion derived from fluorescent microspheres and that obtained using ultrasound flow probes (15, 16).

To the best of our knowledge, renal CT perfusion using the maximum slope method in dogs was evaluated in only our previous study on the effect of the contrast medium injection rate on CT perfusion (17). However, the correlation between CT perfusion analysis and true renal perfusion in dogs remains unknown. Moreover, the effect of a pre-existing contrast medium used for conventional CT scan on CT perfusion parameters has not been investigated. Because both CT studies require a contrast injection, the use of a contrast medium in one study may cause an error in the other study.

We hypothesized that CT perfusion analysis with the maximum slope method can reflect the true renal perfusion in healthy beagles and that the pre-existing contrast medium will not significantly affect the analysis of CT perfusion. The purpose of our study was to evaluate the correlation between CT perfusion derived cortical RBF and the true RBF. Moreover, whether a perfusion analysis can be performed even if CT perfusion is performed after a post-contrast CT scan was evaluated.

## Materials and Methods

The study protocol was approved by the Institutional Animal Care and Use Committee at Chonnam National University. The protocol for the care of dogs adhered to the Guidelines for Animal Experiments of Chonnam National University (CNU IACUC-YB-R-2017-81).

### Animals

This prospective, experimental study used five intact male, purpose-bred beagles. The beagles were obtained from the GENIA corpus (Seoungnam, South Korea) and were raised in the laboratory animal center of Chonnam National University, registered with the Ministry of Food and Drug Safety, for more than 3 months without any other experiments. Dogs were housed in an air-conditioned room in individual cages, and the room temperature was maintained between 23 and 26°C. They were fed with standard commercial dry dog food and tap water *ad libitum*. The median age of the dogs was 3 years (2–4 years), and the median weight was 10.4 kg (9–11 kg). All dogs were clinically healthy based on a physical examination, blood pressure measurement, complete blood count, urinalysis (including urine dipstick and urine specific gravity), serum biochemistry, thoracic and abdominal radiography, abdominal ultrasonography, and echocardiography.

### Perfusion Measurements

After fasting the dogs for 24 h, a 20 G catheter was placed in the cephalic vein and cefazolin sodium (Cefazolin; Chong Kun Dang, Seoul, South Korea) and tramadol hydrochloric acid (Maritrol; Jeil Pharm Co., Seoul, South Korea) were administered intravenously. General anesthesia was induced via an intramuscular injection of a combination of 0.75 mg/kg zolazepam hydrochloride–tiletamine hydrochloride (Zoletil® Virbac, Carros, France) and 0.03 mg/kg medetomidine hydrochloride (Domitor® Orion Corporation, Espoo, Finland). After endotracheal intubation, anesthesia was maintained with isoflurane (1%−2%; Terrell; Piramal Critical Care, Bethlehem, PA, the USA) and oxygen (1 L/min). Then, 0.9% saline solution (CJ Healthcare, Seoul, South Korea) was administered intravenously at a rate of 5–10 mL/kg/h during surgery and CT scanning. A urinary catheter was placed in the bladder to monitor urine output and prevent infection from urination. The dog was monitored by pulse oximetry, electrocardiogram, and Doppler blood pressure measurements from the induction of general anesthesia to recovery.

With the dog in the dorsal recumbent position, the left kidney was exposed via a ventral midline abdominal incision. After separating the renal artery from the surrounding tissues, a 2.0-mm ultrasonic flow probe (2PS Flow probe; Transonic Systems, New York, USA) was placed around the renal artery as close as possible to the renal hilum, and the sliding bracket was closed. The transducer body and the reflector bracket formed the sensing window for the assessment of the renal artery. The cable of the ultrasonic flow probe was plugged into the connector on the flow meter module (TS420; Transonic Systems), and the flow signal quality was monitored based on a bar indicator on the LED digital display of the flow meter module that depicts the received signal amplitude. Simultaneously, the true renal arterial flow (mL/min) was continuously shown on the digital display. After confirming whether the flow signal quality was good and whether the true renal arterial flow was consistently displayed on the flow meter module, the kidney was returned to its original position. To prevent signal quality degradation due to the presence of gas between the probe and renal artery, approximately 50 mL of warm sterilized saline was injected into the abdominal cavity. The abdominal wall and the skin were temporarily closed using continuous sutures with the flow probe cable left outside of the body.

The dog was then moved to the CT examination room while maintaining its position, and the true renal arterial flow was displayed on the flow meter module. In each dog, CT scan was conducted four times in the order of pre-contrast, first CT perfusion, post-contrast, and second CT perfusion. All CT images were acquired using a 16-row multidetector CT scanner (Siemens Emotion 16; Siemens, Forchheim, Germany) while the dogs were in the dorsal recumbent position. A pre-contrast CT scan of the abdomen was conducted with the following settings: slice thickness = 1 mm, pitch = 0.8, rotation duration = 600 ms, tube voltage = 120 kV, and tube current = 120 mA. Then, the first CT perfusion scan was performed via a sequential CT scan at the level of the left renal hilus after injection of 1 ml/kg iohexol (Omnipaque 300; GE Healthcare, Oslo, Norway) at a rate of 4.5 mL/s with a power injector (OptiVantage DH; Mallinckrodt, St. Louis, USA). The CT images were acquired from 10 s after the initiation of contrast injection to 50 s with 4.8-mm coverage using the following settings: rotation time = 1 s, tube voltage = 120 kV, and tube current = 80 mA. Based on the CT perfusion images, the time-to-attenuation curve was drawn by placing a region of interest (ROI) over the left renal cortex. The scan delay for the post-contrast CT scan used to obtain the corticomedullary phase was defined as the time to peak enhancement of the left renal cortex. Then, 10 min after the first CT perfusion scan, post-contrast CT images were obtained after injection of 2 mg/kg iohexol at 4.5 mL/s using a power injector. Then, 2 min after the post-contrast CT scan, the second CT perfusion scan was repeated using the same protocol used in the first CT perfusion scan. Before each CT perfusion scan, the true renal arterial flow displayed on the flow meter module was recorded, and blood pressure was measured using the Doppler ultrasonic flow detector technique.

After completion of the CT scan, the abdomen was reopened, and the flow probe was removed. The abdomen was closed after gross assessment of complications, such as abdominal bleeding and kidney or ureter damage. Until full recovery from anesthesia, the dog's condition was monitored about body temperature, heart rate, blood pressure, and respiratory rate. Postoperative pain was assessed based on activity, posture, appetite, restlessness, crying, and abdominal pressure. Analgesic (tramadol hydrochloric acid 3 mg/kg) and antibiotics (cefazolin sodium 20 mg/kg) were administered *per se* twice a day for 7 days after surgery. The dogs were monitored for complications correlated with surgery for 7 days based on clinical signs including digestive and urinary tract symptoms and changes in heart rate. Body temperature assessment, complete blood count analysis, urinalysis, serum biochemistry, thoracic radiography, abdominal radiography, and abdominal ultrasonography were performed after 7 days.

### Perfusion Analysis

**Figure 1:**
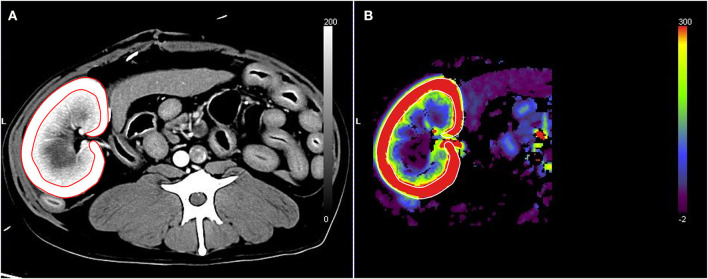
Renal blood flow analysis using computed tomography perfusion images. The region of interest was set by tracing the left renal cortex based on the maximum projection images **(A)**. The blood flow of the left renal cortex was measured in the blood flow map **(B)**.

The CT scan images were sent to a workstation and assessed by a veterinarian (S-KL) with 6 years of radiology experience who was blinded to true RBF of the dogs. CT perfusion was analyzed based on the maximum slope method using the installed CT perfusion software (Syngo Body Perfusion CT, Siemens). On the CT perfusion images, after defining the analysis region, including the whole left kidney and the aorta, motion correction was performed. Then, the arterial input function was determined by drawing a circular ROI on the aorta. For the renal perfusion analysis, the left renal cortex was selected using a threshold value of -50–300 HU. Subsequently, the maximum projection image, blood flow map, and blood volume map were obtained. A tracer ROI was placed over the left renal cortex in the maximum projection images or blood volume map image (Figure 1). The blood flow of the left renal cortex (mL/100 mL/min) was measured three times, and the average value was used for analysis. CT perfusion analysis was conducted three times with at least a 1 week interval by the same reviewer (S-KL) blinded to the previous CT perfusion data and true renal blood flow in the dog, and three CT scan perfusion data were compared for intra-observer reliability assessment. The first measurement was used for further analysis.

**Figure 2:**
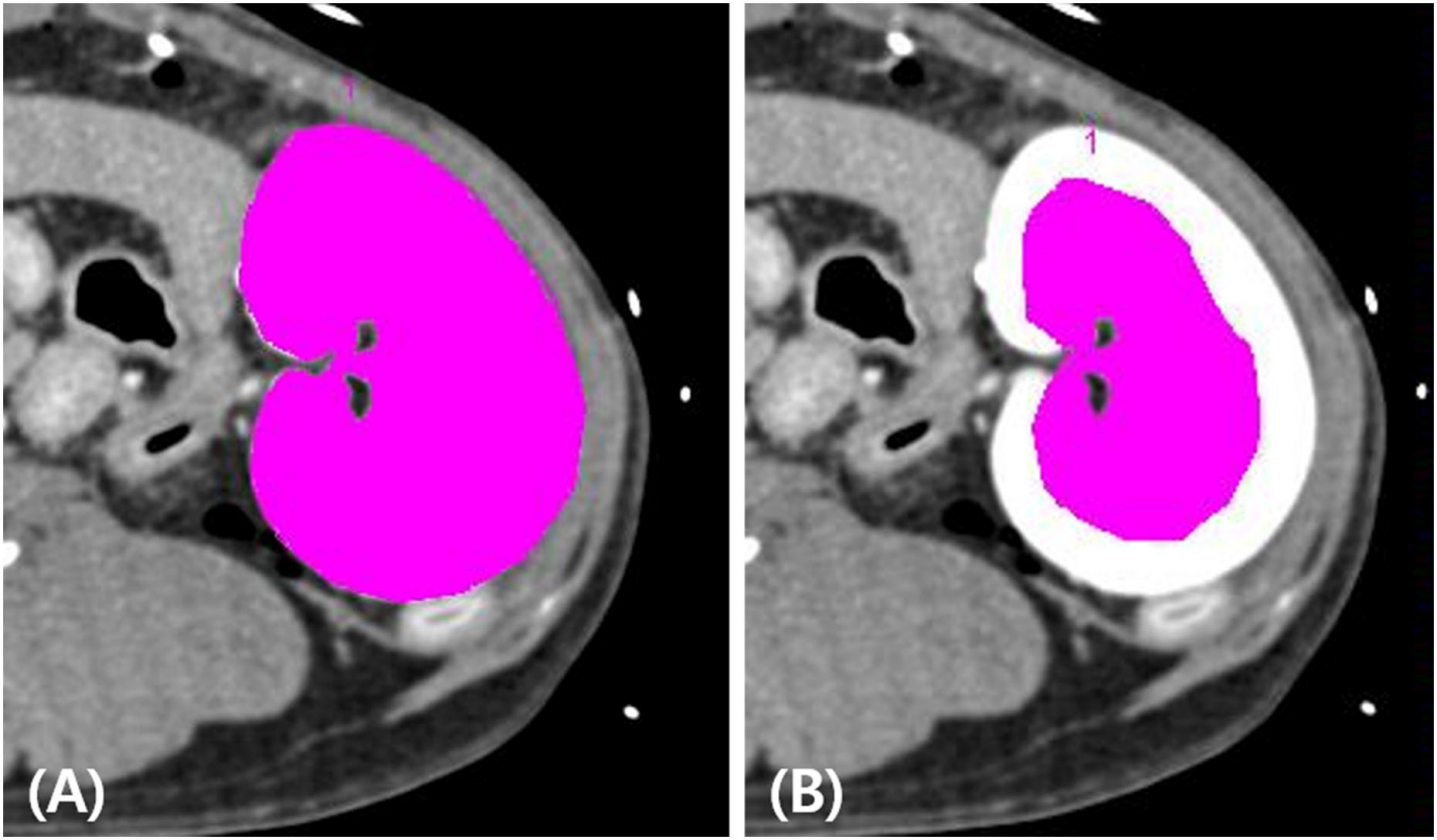
Measurement of the left renal cortex volume on post-contrast computed tomography images. The volumes of the whole left kidney **(A)** and the left renal medulla and pelvis **(B)** were measured by placing the tracer regions of interest with thresholds of 50–1,500 HU using a software tool. The volume of the left renal cortex was calculated by subtracting the volume of the renal medulla and pelvis from the volume of the whole left kidney.

The true RBF was calculated by dividing the true renal arterial flow by the left renal cortical volume. To measure the left renal cortical volume, one ROI was manually traced on the left kidney, and another ROI was drawn to include the medulla and pelvis of the left kidney. The volumes of the whole kidney, renal medulla, and pelvis were automatically calculated using the installed software (Syngo Volume Evaluation, Siemens), with a threshold of 50–1,500 HU (Figure 2). The volume of the left renal cortex was calculated by subtracting the volume of the renal medulla and pelvis from the volume of the entire left kidney. Finally, the ratio of CT perfusion derived RBF to true RBF in each measurement was calculated.

### Statistical Analysis

Statistical analysis was performed using the Statistical Package for the Social Sciences software (IBM SPSS Statistics version 25; IBM, Corporation, NY, the USA). Data are presented as mean and standard deviation. The intra-observer reliability of RBF measurement in CT perfusion images was analyzed by calculating the intraclass correlation coefficient (ICC). Intra-observer reliability was defined as poor if the ICC was <0.4, moderate if it was 0.41–0.6, good if it was 0.61–0.79, and excellent if it was >0.8. Mann–Whitney *U*-test was used to evaluate the difference between CT perfusion derived RBF and the true RBF. Bland–Altman analyses were performed to evaluate the agreement between CT perfusion derived RBF and the true RBF. The mean of difference (bias) and the 95% confidence interval for bias were calculated. Spearman correlation test was used to evaluate the correlation between CT perfusion derived RBF and the true RBF. Wilcoxon signed-rank test was used to evaluate the differences in the true renal arterial flow, true RBF, and CT perfusion derived RBF between the first and second CT perfusion scans. The level of significance was set as a *p* < 0.05.

## Results

The true renal arterial flow measurement and CT perfusion scans were conducted successfully in all dogs. Vocalization and restlessness due to pain were observed during the recovery period. However, pain was well controlled with an analgesic, and the dogs did not present with severe pain during the post-recovery monitoring period. There were no periprocedural and postprocedural complications.

**Figure 3:**
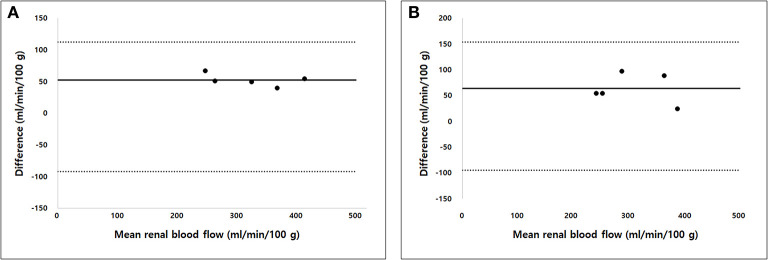
Bland–Altman analysis of the agreement between computed tomography (CT) perfusion and true renal blood flow in the first **(A)** and second **(B)** CT perfusion scan. Bias (solid line) and 95% limits of agreements (dashed lines) are shown.

**Figure 4:**
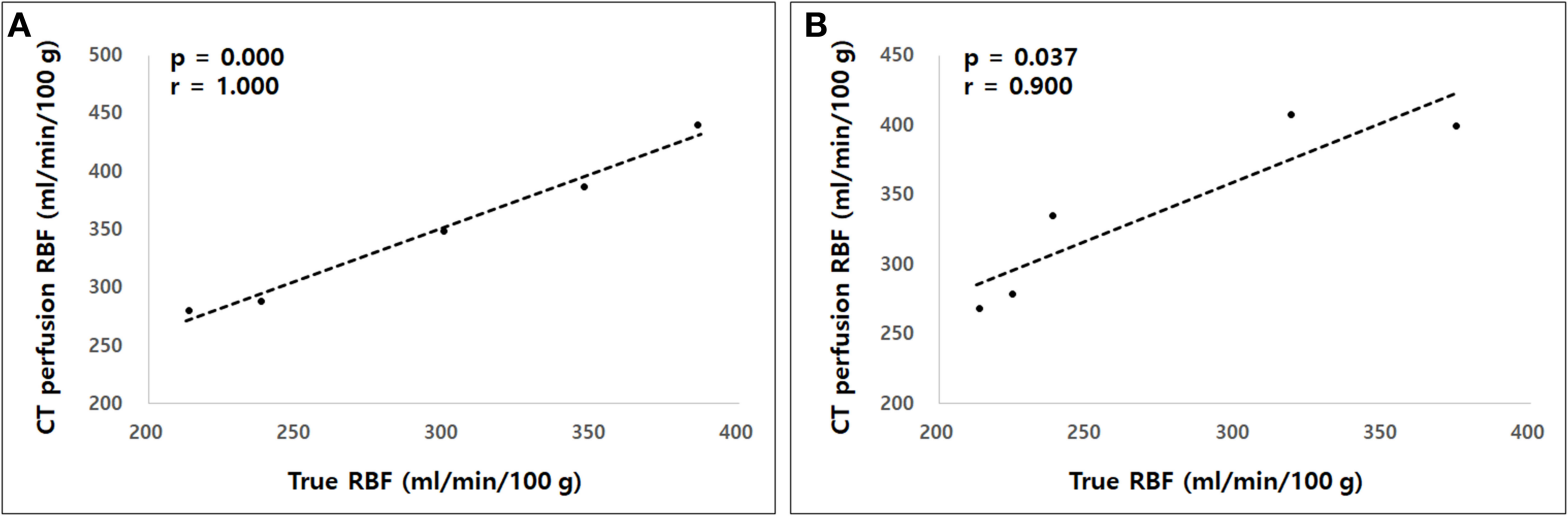
The Spearman correlation test between computed tomography (CT) perfusion derived renal blood flow (RBF) and the true RBF in the first **(A)** and second **(B)** measurements. A strong correlation was observed between CT perfusion derived RBF and the true RBF in both measurements.

The true renal arterial flow values measured using ultrasonic flow probes were 62.50 ± 11.52 and 59.60 ± 4.67 mL/min. The volumes of the whole kidney, renal medulla, and renal cortex were 40.03 ± 7.52, 23.11 ± 3.33, and 18.13 ± 6.23 mL, respectively. The intra-observer reliability of CT perfusion was excellent based on the first (*p* = 0.000, ICC = 0.985) and second (*p* = 0.000, ICC = 0.974) perfusion scans. The true RBF, CT perfusion derived RBF, and the ratio between CT perfusion derived RBF and the true RBF are shown in Table 1. There was no significant difference in the true renal arterial flow (*p* = 0.593), true RBF (*p* = 0.593), and CT perfusion derived RBF (*p* = 0.893) between the first and second CT perfusion scans. CT perfusion derived RBF was not significantly different from the true RBF in the first (*p* = 0.251) and second (*p* = 0.117) CT perfusion scans. As the absolute value of RBF, CT perfusion overestimated the true RBF by approximately 22% in the first and 31% in the second CT perfusion scans. In Bland–Altman analysis, the bias between CT perfusion derived RBF and the true RBF were 52.07 mL/min/100 g, with 95% limits of agreement ranging from −92.15 to 111.95 mL/min/100 g in the first and 63.56 mL/min/100 g, with a 95% limits of agreement ranging from −95.32 to 153.84 mL/min/100 g in the second CT perfusion scan (Figure 3). In Spearman correlation test (Figure 4), CT perfusion derived RBF was found to be significantly correlated with the true RBF in the first (*p* = 0.000, *r* = 1.000) and second (*p* = 0.037, *r* = 0.900) CT perfusion scans.

**Table 1:** Renal blood flow (RBF) measured using an ultrasound flow probe and CT perfusion scan.

	**True RBF**	**CT perfusion derived RBF**	**Ratio of true RBF to CT perfusion derived RBF**
First measurement	273.68 ± 78.94	321.76 ± 62.47	1.21 ± 0.18
Second measurement	246.28 ± 37.45	323.02 ± 48.51	1.31 ± 0.08

Data are presented as mean ± standard deviation.

*The RBF unit is mL/min/100 g*.

## Discussion

This study evaluated whether the RBF derived from CT perfusion could reflect the true RBF measured using an ultrasonic flow probe and whether the previous injection of a contrast medium affected the CT perfusion value in dogs. CT perfusion derived RBF was significantly correlated with true RBF, even in the presence of pre-existing contrast medium in the kidney.

In CT perfusion, RBF can be derived from CT images via post-processing using the maximum slope model or the deconvolution model. In this study, the maximum slope method was used in calculating the perfusion parameters because it is simple and fast to use. Moreover, it is less susceptible to respiratory motion artifact due to the short data acquisition time (16). The maximum slope method is based on the Fick principle, which assumes that contrast medium accumulation in the target organ is equal to the difference in contrast medium concentration between the arterial inflow and venous outflow for that organ (18). Assuming that there is no venous outflow of the contrast medium and no delay from the arterial input to tissue enhancement during perfusion analysis, the accumulation of the contrast medium in the tissue is only determined based on blood flow and arterial input function. Therefore, the blood flow is calculated as the rate of accumulation of the contrast medium in the tissue divided by the degree of arterial contrast enhancement. The rate of contrast accumulation peaks when the arterial concentration reaches to its maximum. Blood flow is the ratio of the maximum slope of increasing tissue enhancement to the maximum arterial concentration. To satisfy the assumption of the maximum slope method, a short contrast injection duration and high contrast medium injection rate are recommended (19, 20). In this study, the contrast medium was injected at a high rate (4.5 mL/s).

In this study, direct flow measurements obtained with an ultrasonic flow probe were used as the reference standard value for blood flow. The ultrasonic flow probe can accurately measure the velocity of fluid using ultrasound to calculate the volume flow. The application of the ultrasonic flow probe technique in RBF measurement in dogs has been validated (21). This technique was used to provide the reference values of RBF in the studies using power-Doppler ultrasonography and CEUS and to evaluate the change in renal perfusion in dogs with renal arterial stenosis and renovascular hypertension (21, 22). After placing the probe to the renal artery, we could continuously measure blood flow with the probe. Thus, renal perfusion data could be obtained simultaneously using both the probe and CT perfusion. Because the flow probe can only measure the renal arterial blood flow (mL/s), the true perfusion value of the left kidney was calculated by dividing the true renal arterial flow by the left renal cortical volume measured on CT scan images.

In this study, the RBF derived from CT perfusion was compared with the true RBF measured directly with an ultrasonic flow probe. To the best of our knowledge, the comparison between CT perfusion based on the maximum slope and the true renal perfusion was performed in only one study using pigs (16). A linear regression analysis showed fairly strong correlation between the perfusion rate derived from CT perfusion scan and that measured using a flow probe in the study. Similarly, although the absolute values of CT perfusion derived RBF and the true RBF were not fully matched, Spearman correlation test showed significant correlation of RBF measured using two methods in our study.

In CT perfusion, blood flow is calculated using computerized image processing and mathematical modeling; thus, there can be differences between CT perfusion and true perfusion. Using the maximum slope method, the perfusion value can be underestimated with a low contrast injection rate and long sampling interval (19, 23). In our study, a sufficiently rapid contrast injection rate and short sampling interval were used to prevent the underestimation of the perfusion value. However, CT perfusion unexpectedly overestimated the true perfusion. Some hypotheses could explain this result. First, the true perfusion values could be underestimated due to an overestimated kidney volume. In our study, the kidney volume was estimated by drawing the ROI on the strongly enhanced renal cortex in post-contrast CT scan images, and the partial volume artifact may cause the overestimation of the kidney volume. Second, the CT perfusion value may be overestimated by underestimating the degree of peak arterial enhancement due to the partial volume effect. Third, renal perfusion may be temporarily changed due to a non-ionic, iodinated contrast medium during CT perfusion scans; however, renal perfusion decreased after the contrast medium injection in humans unlike in our study (24). Finally, the noise of CT perfusion images can contribute to the overestimation of perfusion values like in humans (25). However, although the image noise was not specifically evaluated in this study, we used a sufficiently high current to prevent significant image noise. In addition, the kidneys have a high blood flow and the noise effect was almost non-existent at a higher blood flow (120 mL/100 mL/min) in a previous study (25).

This result indicated that the CT perfusion derived RBF should not be used as the direct compatible parameter to predict the true RBF, because the absolute value was different. Instead, the CT perfusion derived RBF can reflect the true RBF based on the correlation between two methods for estimating RBF. We can expect the CT perfusion derived RBF increases linearly with the true RBF. Therefore, renal perfusion assessed on a CT perfusion scan could be more useful for comparing perfusion in both kidneys and in assessing the serial change in renal perfusion rather than used as the absolute threshold for the determination of renal pathology. In human medicine, CT perfusion is used for evaluating renal function in each kidney, differentiating various renal tumor types based on CT perfusion values, and monitoring response to anti-angiogenic therapy for renal tumors (3–7). While there are currently no CT perfusion studies on renal tumors in dogs, it is possible that CT perfusion may be useful in differentiating renal tumor types in dogs as it is in humans. Furthermore, CT perfusion can be used to understand the physiological change in RBF and the renal function in various renal diseases. In dogs with unilateral urinary obstruction caused by calculi, tumors, and torsion or traumatic kidney injury, CT perfusion can predict the decrease of renal function by comparing it with the contralateral kidney and can determine the appropriate treatment option. In addition, CT perfusion can be helpful in monitoring therapeutic response in dogs with acute renal injury or chronic renal diseases.

In cases where both CT perfusion and post-contrast CT scans are required, CT perfusion can be performed first or after the post-contrast CT scan. When CT perfusion is performed first, it can provide tissue perfusion values and time-to-attenuation curves for determination of scan delay for the contrast CT scans instead of the test bolus. However, it can be challenging to determine the location of the lesions and ROI placement for CT perfusion without post-contrast CT images. Moreover, the veins enhanced by the contrast medium in CT perfusion can make it difficult to distinguish arteries from veins on post-contrast CT (26). These limitations can be overcome by performing CT perfusion after the post-contrast CT scan but, in this case, the major concern is that the pre-existing contrast medium could introduce errors in CT perfusion analysis. However, the RBF derived from the second CT perfusion scan had an excellent correlation with the true RBF in our study. The finding of the present study indicates that CT perfusion derived RBF was correlated with true RBF even in the presence of the pre-existing contrast medium in the kidney injected for another CT study prior to the CT perfusion scan. This result is attributed to the fact that CT perfusion analysis is based on a relative increase in tissue time attenuation regardless of the baseline attenuation (16). Since CT perfusion scan could be performed within <1 min and perfusion data were not affected by the pre-existing contrast media, CT perfusion scan can be used in addition to urinary CT study in dogs.

The present study has several limitations. First, the CT perfusion for RBF was performed under general anesthesia after placing the ultrasonic flow probe, and the study design was not consistent with the clinical condition. The long duration of anesthesia could affect the CT-derived renal blood volume; however, the CT perfusion RBF values were similar to those measured immediately after inducing anesthesia using the same techniques in our previous study (17). Second, this study only included a relatively small number of dogs (*n* = 5) because the procedure used to measure direct RBF was invasive. Third, young adult dogs of the same breed were used in this study. As the patient's weight increases, the volume of the contrast medium required for CT perfusion and injection duration increase, which can affect the perfusion values measured using the maximum slope method. The correlation between CT perfusion and true perfusion can differ in large breed dogs. Fourth, the study was conducted in only healthy dogs. However, considering the invasiveness of assessing true perfusion, it is not possible to compare CT perfusion derived RBF and the true RBF in dogs with renal diseases. Finally, in this study, medetomidine was used for anesthesia of the dog, which might have affected the cardiovascular system and RBF (27). However, this study aimed to compare and assess the correlation between true perfusion values and CT perfusion derived RBF, hence the effects of anesthesia could be disregarded.

## Conclusions

CT perfusion derived RBF is correlated with the true RBF, even with the presence of a pre-existing contrast medium in the kidney. Since CT perfusion can slightly overestimate the true RBF, it could be more useful in the assessment of relative change in renal perfusion on serial CT perfusion studies than in the absolute quantification of RBF. In addition, CT perfusion can be useful in estimating the RBF of each kidney in dogs. Although further studies should be conducted to validate the feasibility of CT perfusion to assess the changes in RBF and renal function in kidney diseases, the results of our study can be used as reference data as they showed the correlation between CT perfusion and true perfusion for RBF in dogs. The results of our study also used to help to configure the CT protocol when perfusion evaluation is required. CT perfusion can be used in addition to the conventional CT protocol for evaluating RBF.

## Data Availability Statement

All datasets generated for this study are included in the article/supplementary material.

## Ethics Statement

The study protocol was approved by the Institutional Animal Care and Use Committee at Chonnam National University. The protocol for the care of dogs adhered to the Guidelines for Animal Experiments of Chonnam National University (CNU IACUC-YB-R-2017-81).

## Author Contributions

S-KL and JC established the hypothesis and experimental design. S-KL, YJ, J-WJ, and HJ performed the surgery for flow probe placement and CT scanning. S-KL analyzed the CT perfusion data and performed the statistical analysis. The manuscript was written by S-KL and was revised by JC. All authors contributed to the article and approved the submitted version.

## Conflict of Interest

The authors declare that the research was conducted in the absence of any commercial or financial relationships that could be construed as a potential conflict of interest.
